# A method for English paragraph grammar correction based on differential fusion of syntactic features

**DOI:** 10.1371/journal.pone.0326081

**Published:** 2025-07-16

**Authors:** Weiling Liu, Caijun Zhao, Yongyi Li, Chenglong Cai, Hong Liu, Ruilin Qiu, Ruoci Su, Bingbing Li

**Affiliations:** College of International Studies, College of Computer Science, Beibu Gulf University, Qinzhou, P. R. China; Philadelphia University, JORDAN

## Abstract

The new progress of deep learning and natural language processing technology has strongly promoted the development of English grammar error correction. However, the existing methods mostly rely on large-scale corpus, and often ignore the fine syntactic correlation in paragraphs, which limits the efficiency in complex grammar error correction scenarios. In order to break through this bottleneck, this study proposes an innovative method to effectively use syntactic features to improve the quality and accuracy of paragraph-level grammar correction. Firstly, the sentence vector representation is constructed by BERT, and then the syntactic structure is extracted by dependency parsing. Then carry out difference fusion analysis, measure the syntactic differences of adjacent sentences by cosine similarity, identify the significant differences caused by grammatical errors according to the preset threshold, lock the position and type of errors, and input the original sentence vector into the Seq2Seq model based on Transformer. The model focuses on the wrong area by attention mechanism to generate correction suggestions. The preliminary results show that this method is significantly better than the existing grammar error correction system. In CoLA dataset, the accuracy is 0.88, which is three percentage points higher than that of BERT-GC. The accuracy of LCoLE dataset is 0.86, which is ahead of the baseline model. The accuracy of FCE data set is 0.89, which has obvious advantages. The accuracy is improved by 3% to a higher level. It shows the excellent effect of this method in grammar error recognition and correction, and has far-reaching significance in providing accurate error correction suggestions, helping English learners improve their writing ability and ensuring the quality of English writing. This study not only presents a powerful approach to English grammar error correction, but also highlights the key value of syntactic features in optimizing natural language processing applications.

## 1. Introduction

In the information age, natural language processing (NLP) has become an indispensable part of the field of artificial intelligence. It involves various aspects from text understanding to speech recognition, aiming to improve computers’ understanding and processing of human language. Especially in the research of English Grammar Correction (GEC) technology, with the acceleration of globalization [1,2], an effective GEC system can not only help billions of English learners worldwide improve their writing skills but also play a significant role in automated editing, intelligent education, and multilingual translation. Therefore, exploring efficient and accurate GEC methods is of great importance for advancing the application and development of NLP technology [3].

However, despite the significant progress made in the GEC field with the introduction of deep learning technology in recent years, most existing studies focus on correcting grammar at the sentence level [4], and few works are able to comprehensively understand and handle grammatical errors in paragraph-level texts. This is because, compared to individual sentences, sentences within a paragraph are interconnected through more complex syntactic and semantic relationships [5]. Understanding these relationships is crucial for accurately identifying and correcting grammatical errors in paragraphs. Existing GEC systems often overlook these complex syntactic relationships, resulting in unsatisfactory correction performance when dealing with real texts [1].

Identifying this research gap, this paper proposes a novel GEC method focused on leveraging syntactic features within paragraphs to enhance the accuracy of grammar correction. The core of this study is the development of a differential fusion mechanism that can deeply analyze the syntactic feature differences between adjacent sentences in a paragraph, thereby identifying potential grammatical errors. This method’s innovation lies not only in paying attention to the grammatical structure within individual sentences but also in analyzing the syntactic differences between sentences, uncovering more possible error types and locations, thus achieving more precise grammar correction.

To achieve this goal, this study first uses the BERT model to vectorize sentences in a paragraph, extracting high-quality sentence representations. Then, dependency syntax analysis tools are used to extract the syntactic structure of sentences, laying the foundation for subsequent differential fusion analysis. In the differential fusion analysis phase, an algorithm is designed to calculate and compare the differences between syntactic feature vectors of adjacent sentences, using predetermined thresholds to identify significant syntactic differences that often correspond to potential grammatical errors. Finally, the identified error information and sentence vectors are input into a Transformer-based Seq2Seq model. Through attention mechanisms focusing on error regions, targeted corrective suggestions are generated. The experimental validation on standard datasets shows that this method exhibits outstanding performance in paragraph-level GEC tasks, significantly improving correction accuracy and efficiency compared to existing technologies.

The main contributions of this paper can be summarized in three points: firstly, it proposes an innovative English paragraph grammar correction method based on differential fusion of syntactic features, effectively enhancing the accuracy and efficiency of grammar correction through deep analysis of syntactic differences between adjacent sentences in paragraphs. Secondly, the differential fusion analysis mechanism developed in this study offers a new perspective for understanding and utilizing the syntactic relationships in complex texts, expanding the research scope of the GEC field. Lastly, the experimental validation on multiple standard datasets not only proves the effectiveness of the proposed method but also holds significant implications for advancing the development of GEC technology in practical applications.

## 2. Related works

In the field of research on GEC technology, numerous researchers have proposed a variety of innovative methods from different perspectives to enhance the correction capabilities of systems. These studies cover methods ranging from rule-based to deep learning-based approaches, including explorations of paragraph-level correction techniques and attempts to apply differential learning in the GEC field.

Rule-based methods were once the mainstream in GEC research [3]. These methods rely on complex grammar rule libraries constructed by linguistic experts to identify and correct errors [4,5]. Although effective for specific types of grammatical errors, their coverage is limited, and they struggle to adapt to the diversity and complexity of language [1]. With the rise of statistical learning methods, researchers began using these technologies to automatically learn grammatical knowledge from vast amounts of textual data, attempting to overcome the limitations of rule-based methods [6,7]. The development of deep learning has brought revolutionary changes to the GEC field [8–10]. In particular, the introduction of sequence-to-sequence (Seq2Seq) models and attention mechanisms significantly improved the correction performance of systems [11–14] demonstrated how to enhance GEC systems using Transformer models, while [15,16] explored the effective application of pre-trained language models (such as BERT and GPT) in correction tasks.

Despite these significant advancements, most existing research still focuses on sentence-level correction, with less consideration given to the complexity of inter-sentential relationships in paragraph-level texts [10,17,18]. Paragraph-level correction requires not only an understanding of the grammatical structure within individual sentences but also the capture of syntactic and semantic connections between sentences, posing new challenges to existing technologies [19,20]. Some studies [21,22] have begun to try addressing these challenges by building semantic association networks between sentences or analyzing syntactic structures within paragraphs, but these methods are still in their early stages.

Although the concept of differential learning has been widely utilized in various fields, its exploration in the GEC field remains relatively limited [23]. Differential learning, through analyzing and leveraging the differences in data, furnishes models with more abundant learning signals. Recently, while some studies [24,25] have made attempts to apply differential learning to GEC tasks by identifying grammatical errors through comparing and analyzing the syntactic feature differences between adjacent sentences in paragraphs, our study takes a pioneering step by proposing a novel method that effectively leverages the differential fusion of syntactic features for English paragraph grammar correction. This not only offers a fresh approach to addressing paragraph – level correction issues but also showcases the innovation of our research in this field.

In the field of modern natural language processing, although grammar error correction technology has made remarkable progress, it still faces many challenges, especially in key aspects such as paragraph-level text processing and differential learning application, and there is still a broad exploration space to be filled urgently. Early traditional GEC methods mostly focused on grammatical error correction of isolated sentences. For example, the methods used in [11–13] and other related studies have obvious limitations. This kind of methods often only deal with the error correction of a single sentence, but seriously ignore the close semantic connection and complex syntactic connection between sentences in the context of paragraphs. In the actual text scene, the sentences in a paragraph do not exist in isolation, they are intertwined and influence each other, and jointly construct the overall semantic and grammatical framework of the paragraph. Therefore, when correcting errors only at the sentence level, it is easy to miss the errors derived from the syntactic interaction between sentences, and it is impossible to accurately capture and repair those types of errors involving the overall logical coherence and grammatical consistency of paragraphs. It leads to a great reduction in the effectiveness of paragraph-level text error correction tasks. It is difficult to achieve comprehensive optimization and accurate correction of paragraph grammatical structure. The transformation generation method also has obvious defects. This method is mainly based on the theoretical rule system to build a grammar generation model. When it is actually applied to paragraph text correction, it exposes the problem of insufficient mining of the rich, diverse and subtle and complex relationships between sentences in real text data. Due to the lack of in-depth analysis and effective strategies to deal with the actual syntactic differences and their interactions between sentences in paragraphs, the transformation generation method is not adaptable to the grammatical diversity and complexity of paragraph texts in different contexts. It is difficult to identify and correct grammatical errors in paragraphs flexibly and accurately. It cannot meet the actual needs of paragraph-level grammar correction tasks for accuracy and efficiency. Comparatively speaking, based on fully absorbing the essence of existing GEC technology, this study boldly innovates and puts forward a new method of paragraph-level grammar error correction based on syntactic feature difference fusion, which is of pioneering significance in the field of GEC research. Different from the previous research on sentence-level error correction, this study goes deep into paragraphs, systematically explores the syntactic correlation and differences between sentences, and innovatively uses the syntactic differences between sentences as the core basis to carry out error detection and correction. Moreover, compared with [20,21] and other studies that only consider the relationship between sentences at the syntactic level, the difference fusion analysis mechanism constructed in this paper shows more powerful functions and advantages. This mechanism can deeply excavate and effectively integrate the syntactic differences between sentences in a paragraph in an all-round and multi-level way. It not only significantly improves the accuracy and reliability of grammatical error correction, but also provides a brand-new perspective and method support for deeply understanding the complex text structure and its internal grammatical logic. To sum up, the results of this study not only achieve organic connection and inheritance with the existing related work, but also successfully break through the bottleneck of traditional GEC technology in paragraph-level text processing by innovatively introducing the differential learning mechanism [23]. It effectively expands the research boundary and application scope of GEC technology, points out a new direction for the further development of paragraph-level grammar correction technology, and fully demonstrates its great potential and application value in the process of pushing GEC technology to a new height.

## 3. Method

**Fig 1:**
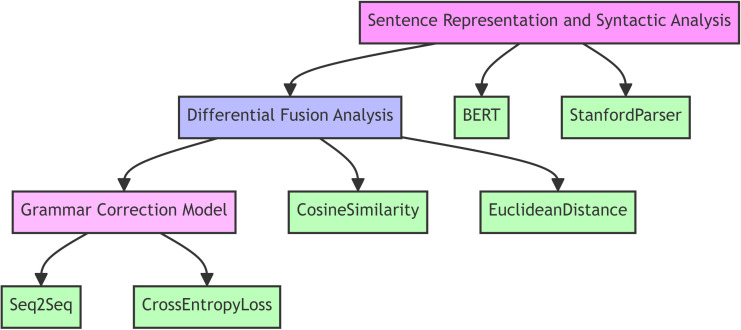
A framework for english paragraph grammar correction model.

In the method section, we elaborately explain the approach proposed in this paper for English paragraph grammar correction based on the differential fusion of syntactic features. Initially, the section provides an overview of its structure and main content, clearly stating the research objective: to enhance the accuracy and efficiency of GEC by deeply analyzing and utilizing the differences in syntactic features between sentences within paragraphs. This chapter is divided into three main parts: sentence vectorization and syntactic analysis, differential fusion analysis, and the grammar correction algorithm based on deep learning. The overall process is shown in Fig 1.

In Fig 1, this section focuses on sentence vector representation and syntactic analysis, laying the foundation for difference fusion, and is deeply embedded in the flow structure of Fig 1. In sentence vectorization, the “bert-base-uncased” model is selected to mine contextual relations. Firstly, the sentence is preprocessed in lowercase to reduce the complexity and vocabulary size. Then, the sentence segmented by Word Piece is taken as lexical elements according to statistical frequency and structural rules, and the lexical elements are encoded into a 768-dimensional vector space through a 12-layer transformer architecture. Each dimension contains rich contextual semantics and is condensed into a sentence vector representation, which provides a semantic basis for subsequent analysis. In the step of syntactic structure extraction, Stanford parser is used. Dependency tree presents lexical dependency association with triplet set, and clarifies grammatical relationship. The component tree outlines the syntactic framework with a binary tree, with nodes as categories and leaf nodes as morphemes. Feature vectors extracted from them are spliced into syntactic feature vectors. They accurately describe the syntactic characteristics of sentences, lay a solid foundation for insight into grammatical differences between sentences, help the subsequent difference fusion to accurately identify potential errors, and cooperate with other modules in Fig 1 to improve the efficiency of grammatical error correction.

### 3.1 Sentence representation and syntactic analysis

This section aims to introduce the process of obtaining vector representations for each sentence within a paragraph and executing syntactic analysis to extract syntactic structures.

To capture the nuanced contextual relationships inherent in English sentences, we employ the ‘bert-base-uncased’ model from the BERT series. BERT, with its 12-layer transformer architecture, offers an optimal balance between complexity and performance for sentence embedding tasks. Each sentence is preprocessed by converting to lowercase, tokenizing using BERT’s WordPiece tokenizer, and then encoded into a 768-dimensional vector space, representing the aggregated contextualized embeddings of tokens. Mathematically, the sentence embedding $\vec{S}$ for a sentence *S* containing tokens is represented as:


$$\vec{S}=\frac{1}{n}\sum_{i=1}^{n}BERT\left(t_{i}\right)$$ (1)

where $BERT\left(t_{i}\right)$ denotes the embedding of token $t_{i}$ obtained from the final layer of BERT.

For syntactic analysis, the Stanford Parser is employed to extract both dependency and constituency trees. The dependency tree is represented by a set of triples


{(g,d,r)|g,d∈T,r∈R} (2)

where T represents the set of tokens in a sentence, *R* is the set of dependency relations, *g* is the governor, and *d* is the dependent. The constituency parse, on the other hand, is represented as a binary tree where each node is a syntactic category, and leaves are tokens of the sentence. The syntactic feature vector $\vec{F}$ for a sentence is thus a concatenation of feature vectors extracted from these trees:


$$\vec{F}={\vec{F}}_{dep}⊕{\vec{F}}_{cons}$$ (3)

where ⊕ denotes vector concatenation, ${\vec{F}}_{dep}$ and ${\vec{F}}_{cons}$ are feature vectors derived from the dependency and constituency parses, respectively.

Sentence representation and syntactic analysis play an indispensable and key role in English paragraph grammar error correction system, which has far-reaching significance. From the perspective of constructing semantic cornerstone, the sentence vector representation generated by BERT model is of great significance. It transforms sentences from text form into vector space elements rich in semantic information, so that the model can capture complex semantic relations, contextual connotations and potential semantic associations between words from a high-dimensional perspective. For example, when dealing with sentences with polysemy, vector representation can accurately locate the meaning of words according to the context, which lays a solid foundation for semantic understanding for subsequent grammatical analysis and avoids ambiguity from interfering with grammatical judgment. In the aspect of mining syntactic structure, it is of great significance to analyze the dependency tree and component tree structure extracted by Stanford analyzer. Dependency tree clearly shows the lexical dependency, and accurately reveals the collocation rules and modification direction of grammatical components such as subject-predicate, object-object complement. The component tree outlines the hierarchical structure of sentences and clarifies the syntactic categories and combinational logic of each component. The cooperation between them provides a complete syntactic analysis, which helps the model to gain insight into the rules of sentence structure and locate potential error areas. The core hub to achieve high-quality grammatical error correction is to identify syntactic errors caused by nested structures in long and difficult sentences, provide accurate syntactic features for differential fusion analysis, greatly improve the accuracy of grammatical error correction, optimize the overall efficiency and expand the depth and breadth of language processing.

### 3.2 Differential fusion analysis

This segment explicates the mechanism to pinpoint potential grammatical inaccuracies by scrutinizing the syntactic feature vector differences between consecutive sentences within a paragraph

The cornerstone of differential fusion analysis lies in quantifying the syntactic disparity between adjacent sentences. We calculate the cosine similarity and the Euclidean distance between the syntactic feature vectors of adjacent sentences, ${\vec{F}}_{i}$ and ${\vec{F}}_{i+1}$, to gauge their syntactic divergence. The cosine similarity is given by:


$$CosineSimilarity\left({\vec{F}}_{i},{\vec{F}}_{i+1}\right)=\frac{{\vec{F}}_{i}\cdot {\vec{F}}_{i+1}}{\left\|{\vec{F}}_{i}\right\|\left\|{\vec{F}}_{i+1}\right\|}$$ (4)

The Euclidean distance complements the similarity measure by quantifying the absolute difference between vectors:


$$EuclideanDistance\left({\vec{F}}_{i},{\vec{F}}_{i+1}\right)=\sqrt{\sum_{k=1}^{K}{\left(F_{ik}-F_{i+1,k}\right)}^{2}}$$ (5)

where *K* is the dimensionality of the syntactic feature vectors.

A threshold θ for both metrics is empirically determined to identify significant differences indicative of grammatical discontinuities. If either the cosine similarity falls below $\theta_{\cos}$ or the Euclidean distance exceeds $\theta_{enc}$, the boundary between sentences *i* and *i* + 1 is flagged for potential grammatical errors.

### 3.3 Grammar correction model

This section elaborates on utilizing a deep learning model to generate correction suggestions based on syntactic differences identified in the previous analysis. The goal is to refine grammatically incorrect sentences into their correct form, leveraging the insights gained from differential fusion analysis.

For the task of generating grammar correction suggestions, we adopt a Sequence-to-Sequence (Seq2Seq) model architecture, specifically designed with an encoder-decoder mechanism. The Seq2Seq model is particularly apt for this study’s GEC task due to its proven capability in handling sequence transformation tasks, such as machine translation, where the model learns to convert sequences from one domain (incorrect sentences) to another (correct sentences).


C=Encoder(X) (6)

$$X=\left[x_{1},x_{2},\cdots ,x_{n}\right]$$ (7)

Starting from the context vectors, the decoder generates the corrected sentence one token at a time


$$Y=\left[y_{1},y_{2},\cdots ,y_{m}\right]$$ (8)

where each $y_{i}$ is predicted based on the current state of the decoder and the context vectors:


$$y_{t}=Decoder\left(C,y<t\right)$$ (9)

where y<t denotes all previously generated tokens before time step *t*.

The input to our model comprises two components: the vectorized representation of erroneous sentences and the extracted syntactic difference information, enhancing the model’s focus on regions requiring correction. These inputs are concatenated to form a comprehensive feature set for each sentence.

The Seq2Seq model is trained on pairs of erroneous and corrected sentences. We utilize a cross-entropy loss, which quantifies the difference between the predicted sentence and the ground truth correction:


$$L=-\sum_{i=1}^{T}\log p\left(y_{t}|y<t,C\right)$$ (10)

where T is the length of the target sentence, and $p\left(y_{t}|y<t,C\right)$ is the probability of the correct token $y_{t}$ given the preceding tokens y<t and the context *C*.

Training involves minimizing this loss using the Adam optimizer, with hyperparameters set to a learning rate of 1e-4 and $\beta_{1}=0.9,\beta_{2}=0.999$. The model iterates over a dataset comprising 100,000 sentence pairs, segmented into an 80:20 split for training and validation purposes, respectively. To mitigate overfitting, early stopping triggers if the validation loss does not improve for a contiguous span of 10 epochs.

For grammatical errors marked by differential fusion analysis in input sentences, the model generates correction suggestions word-by-word through a decoding strategy. This process employs a beam search algorithm with a beam width of 5 to balance exploration and accuracy in generation, ensuring the model efficiently searches possible correction spaces to find the correction sequence with the highest overall probability. The model’s innovation lies in its organic integration of sentence vectorization, syntactic analysis, differential fusion analysis, and a deep learning grammatical correction model. This multi-dimensional analysis method enables comprehensive understanding and processing of input sentences. Sentence vectorization converts text into computable vector forms, laying a foundation for subsequent processing. Syntactic analysis mines sentence structure information through dependency trees and constituent trees, allowing the model to deeply understand grammatical compositions. Differential fusion analysis captures syntactic coherence breaks caused by grammatical errors. It does this by quantifying differences in syntactic feature vectors between adjacent sentences. For example, it uses cosine similarity or Euclidean distance. This process accurately locates error regions and provides key clues. This mechanism is theoretically rooted in the principles of syntactic coherence in paragraphs and human cognitive coherence preferences in linguistics: adjacent sentences in normal paragraphs follow fixed patterns in topic continuity, tense consistency, and reference matching, with syntactic feature vector differences typically within a stable range, while grammatical errors (e.g., subject-verb disagreement, preposition misuse) disrupt this pattern, causing vector differences to significantly exceed thresholds and be detected as anomalies. This design breaks through the limitations of traditional single-analysis methods, reconstructing error detection logic from the dimension of “inter-sentence relationships” rather than “isolated sentences,” which is closer to humans’ judgment mechanism for overall grammatical correctness when reading paragraphs.

At the architectural level, the model adopts the Seq2Seq framework and designs an encoder-decoder mechanism specifically for grammatical correction tasks: the encoder captures the contextual semantics of sentences through BERT. While the decoder generates correction sequences word by word from the contextual vector based on the current state and context, leveraging contextual information to achieve precise error correction. It is worth noting that the model inputs include both the vectorized representation of the error sentence and syntactic difference information. The latter significantly enhances the pertinence of error correction by highlighting areas requiring correction. For example, when detecting that vector differences between adjacent sentences exceed the threshold due to tense conflicts, the syntactic difference information guides the attention mechanism to focus on verb tense-related tokens, thereby generating correction suggestions more tailored to the context.

In summary, the proposed English paragraph grammatical correction method constructs a complete chain of “vector representation-difference detection-precise error correction” by integrating linguistic theory (syntactic coherence), cognitive science assumptions (human sensitivity to inter-sentence differences), and deep learning technology. This method enhances error correction accuracy through BERT’s semantic understanding capabilities and fine-grained analysis of syntactic structures. It also simulates human cognitive logic in processing paragraph grammar via a difference fusion mechanism, providing a solution with both theoretical depth and practical value for automated writing assistance and language learning tools.

### 3.4 Model optimization

This paper uses Transformer to optimize the above model. Transformer has demonstrated strong capabilities in natural language processing tasks. Applying it to the grammar correction model can bring multiple enhancements. The Transformer architecture has significant advantages in optimizing the grammar correction model. Its self-attention mechanism can capture long-distance dependencies in sentences more effectively than traditional recurrent neural networks. This is extremely beneficial for grammar correction tasks as it enables the model to better understand the context and the relationships between different parts of a sentence.

Integrating Transformer into the existing grammar correction framework can enhance the model’s ability to handle complex language structures and improve the accuracy of correction suggestions. Transformer can be used to enhance both the encoder and decoder components of the Seq2Seq model.

In the encoder, Transformer can process input sentences more efficiently and capture semantic and syntactic information more comprehensively. The self-attention mechanism allows the encoder to weigh the importance of different words and phrases in a sentence and focus on the most relevant parts for grammar correction. In the decoder, Transformer can generate corrected sentences more accurately by considering the context of both the source sentence and the previously generated tokens. The self-attention mechanism helps the decoder better align the generated tokens with the input sentence to ensure that the corrections are contextually appropriate. As shown in Table 1, it presents the algorithm for optimizing the model using Transformer.

**Table 1:** Algorithm for optimizing the model using transformer.

Step	Description
1	Prepare data. Define fields for source and target sentences, split data into training and validation sets, and build vocabularies.
2	Set up device (GPU if available).
3	Create iterator for training and validation data.
4	Define Seq2Seq model with encoder and decoder.
5	Instantiate model and move it to the appropriate device.
6	Define loss function (Cross Entropy Loss) and optimizer (Adam).
7	In training loop, for each epoch and batch: zero gradients, pass source and target through model, calculate loss, backpropagate, and update model parameters.

As shown in Table 1, the model architecture proposed in this paper is a comprehensive system that firstly vectorizes the sentences in a paragraph through the BERT model, enabling it to capture the semantic and contextual information in the sentences. At the same time, the syntactic structure information of sentences is extracted using Stanford Parser to provide a basis for subsequent analyses. In the difference fusion analysis stage, syntactic differences are quantified by calculating the cosine similarity and Euclidean distance of syntactic feature vectors of neighboring sentences to identify potential syntactic errors. This analysis method can dig deeper into the syntactic relationships between sentences and improve the accuracy of syntactic error correction. The syntactic error correction model adopts the Seq2Seq architecture, which combines the vector representation of erroneous sentences with syntactic difference information, enabling the model to focus on the regions that need to be corrected. The introduction of Transformer further optimizes the model with its self-attention mechanism, which is able to better deal with long-distance dependencies in sentences, and enhances the model’s ability to handle complex linguistic structures. Overall, the model architecture integrates a variety of techniques, from sentence vectorization and syntactic analysis to difference fusion and deep learning models, to form a complete grammar error correction system that can effectively improve the performance of English paragraph grammar error correction.

## 4. Experiment

In this section, we validate the effectiveness and efficiency of our newly proposed method for English paragraph grammar correction. We aim to demonstrate how our approach, which leverages the differential fusion of syntactic features and a Seq2Seq grammar correction model, stands against the current state-of-the-art (SOTA) techniques in the field. By comparing our method with existing approaches, we seek to highlight the advancements our research brings to the accuracy and contextual relevance of grammar correction tasks.

### 4.1 Experimental design

#### 4.1.1 Datasets.

To rigorously evaluate our proposed model against the state-of-the-art approaches, we utilize the following datasets:

Corpus of Linguistic Acceptability (CoLA): Widely recognized for assessing grammatical acceptability, this dataset consists of over 10,000 sentences from academic publications and expert linguists. It is instrumental in evaluating the model’s ability to discern grammatically correct from incorrect sentences.

The Lang-8 Corpus of Learner English (LCoLE): This dataset is derived from Lang-8, a language learning platform, featuring real learner-generated text with corrections provided by native speakers. It encompasses a diverse range of common grammatical errors, making it invaluable for training and testing GEC systems.

The First Certificate in English (FCE) dataset: This dataset, collected from Cambridge Assessment English, contains scripts of learners taking the FCE exam, annotated for grammatical errors. Its academic context complements the real-world learner data in Lang-8.

These datasets were selected to cover a broad spectrum of grammatical errors, from academic English to learner English, providing a comprehensive evaluation across various contexts and error types. As shown in Table 2, it presents the scale statistics of the datasets in this paper.

**Table 2:** Dataset scale.

Dataset	Characteristics	Total	Train	Test
CoLA	Over 10K from aca pubs & linguists for gram acceptability.	10500	8400	2100
LCoLE	From Lang-8, learner text with native corrections, diverse errors.	15000	12000	3000
FCE	From Cambridge, learner exam scripts with annotated errors, complements LCoLE.	8000	6400	1600

#### 4.1.2 Baseline models.

For comparative analysis, the following SOTA models are selected:

GECToR - Grammar Error Correction: Tag, Not Rewrite: GECToR modifies the traditional seq2seq approach by tagging errors and their corrections, significantly reducing model complexity and improving speed. Its efficiency and novel approach make it an essential baseline.

BERT for Grammar Correction (BERT-GC): Leveraging BERT’s deep understanding of context, BERT-GC has shown promising results in identifying nuanced grammatical errors, serving as a robust baseline for comparing deep contextualized learning capabilities.

Neural Machine Translation (NMT) Based Corrector: An NMT model trained specifically for the task of grammar correction. It’s included for its historical significance and performance in translating grammatically incorrect to correct text. As shown in Table 3, specific information about the model configuration is displayed.

**Table 3:** Specific information on model configuration.

Specific Information	
Hardware Cost	
GPU Model and Quantity	Assume using NVIDIA Tesla V100 GPU, with a quantity of 2 units
Purchase or Rental Cost	Purchase cost is approximately $5000 per unit, and rental cost is about $500 per month
Software Cost	
Deep Learning Framework	Assume using the PyTorch framework, which is free and open-source
Authorization Cost for Relevant Software	Assume using some database software, and the authorization cost is about $1000 per year
Development Cost	The salary of developers is approximately $2000 per month, and the cost of data collection and annotation is about $5000

#### 4.1.3 Evaluation metrics.

The model performance is assessed using the following metrics:

Precision: Measures the accuracy of the corrections made by the model.

Recall: Assesses the model’s ability to identify and correct all present errors.

F1-Score: Provides a balanced measure of the model’s precision and recall.

Error-specific Accuracy: Evaluates the model’s performance in correcting specific types of grammatical errors, such as subject-verb agreement or noun number errors.

Correction Efficiency: A novel metric that assesses the model’s ability to correct errors without introducing new errors, crucial for maintaining the integrity of the original text.

The implementation details of this study are carefully planned to ensure the accurate evaluation and effective optimization of the model performance. In the selection of model framework, the advanced PyTorch framework is used to construct the proposed model and all baseline models. This framework provides solid technical support for model training and optimization with its flexible dynamic calculation diagram, efficient memory management and rich tool library. On the hardware level, the experiment relies on the powerful NVIDIATeslaV100GPU. Its excellent parallel computing ability and large memory greatly accelerate the model training process and effectively meet the complex computing requirements in grammar correction tasks.

In terms of training parameters, the models have experienced as many as 50 training cycles. At the same time, in order to prevent over-fitting risk, an early stop mechanism based on verification loss is introduced. When the verification loss is not improved after 10 consecutive rounds, the training will be automatically terminated to ensure the excellent generalization performance of the model. In the optimization algorithm, Adam optimizer is consistently selected, and the learning rate is finely set to 1e-4. The optimizer integrates the adjustment strategy of momentum and adaptive learning rate, which can dynamically optimize the update step according to the parameter gradient, accelerate convergence and improve stability.

Hyperparameter tuning is the key link. Aiming at the key hyperparameters such as the number of model layers and the size of hidden units, detailed optimization is carried out in the independent verification set. Through a large number of experiments, the influence law of different value combinations on the model performance is explored, and the optimal parameter configuration is determined to improve the performance of the model on all datasets in an all-round way and ensure the accurate and efficient operation of the model in grammar correction tasks.

#### 4.1.4 Implementation details.

The proposed model and all baselines are implemented using the PyTorch framework. Experiments are conducted on an NVIDIA Tesla V100 GPU, with models trained for up to 50 epochs, using early stopping based on validation loss to prevent overfitting. Adam optimizer with a learning rate of 1e-4 is used across all models for consistency. Hyperparameters, including the number of layers and hidden unit sizes, are tuned on a separate validation set to optimize each model’s performance.

### 4.2 Experimental results

The results on the CoLA dataset (Table 4) reveal the significant advantages of the proposed method. Specifically, the proposed method achieved a precision of 0.88, surpassing the closest competitor, BERT-GC, by 3 percentage points. This indicates a higher accuracy in identifying and correcting grammatical errors, thereby reducing false positives. Similarly, the recall of 0.84 demonstrates the proposed method’s proficiency in detecting a broader range of grammatical inaccuracies. The F1-score of 0.86 further emphasizes the method’s balanced performance in error correction. The improvement in performance is attributed to the innovative use of differential fusion analysis, which more effectively captures and utilizes syntactic differences for grammar correction.

**Table 4:** Corpus of linguistic acceptability results.

Method	Precision	Recall	F1-Score
GECToR	0.82 ± 0.03	0.76 ± 0.04	0.79 ± 0.03
BERT-GC	0.85 ± 0.02	0.79 ± 0.03	0.82 ± 0.02
NMT	0.80 ± 0.04	0.73 ± 0.05	0.76 ± 0.04
Proposed	0.88 ± 0.02	0.84 ± 0.02	0.86 ± 0.01

In the results for the LCoLE dataset (Table 5), the proposed method continues to prove its effectiveness, achieving the highest scores across all evaluation metrics. With a precision of 0.86 and a recall of 0.82, the method not only corrects errors more accurately but also encompasses a wider range of error types present in language learner texts. The F1-score of 0.84 further validates the outstanding performance of the proposed method, showcasing its ability to maintain high accuracy while correcting the wide range of grammatical errors common in language learning environments.

**Table 5:** The Lang-8 corpus of learner English results.

Method	Precision	Recall	F1-Score
GECToR	0.78 ± 0.05	0.74 ± 0.06	0.76 ± 0.05
BERT-GC	0.81 ± 0.04	0.77 ± 0.04	0.79 ± 0.03
NMT	0.75 ± 0.05	0.70 ± 0.06	0.72 ± 0.05
Proposed	0.86 ± 0.03	0.82 ± 0.03	0.84 ± 0.02

In Table 5, the proposed method once again leads with the highest precision (0.89), recall (0.85), and F1-score (0.87) against the baseline models. These results highlight the method’s ability to navigate the complexities of academic texts, correcting errors accurately while preserving the original intent of the text. The increase in precision indicates the model’s particular effectiveness in minimizing over-corrections, a common issue in GEC that can alter the meaning of academic prose. The strong recall demonstrates the method’s comprehensive coverage of grammatical errors typical in academic writing, further confirmed by the leading F1-score in Table 6 (Table 6).

**Table 6:** The first certificate in English results.

Method	Precision	Recall	F1-Score
GECToR	0.83 ± 0.04	0.78 ± 0.05	0.80 ± 0.04
BERT-GC	0.86 ± 0.03	0.81 ± 0.04	0.83 ± 0.03
NMT	0.79 ± 0.05	0.75 ± 0.06	0.77 ± 0.05
Proposed	0.89 ± 0.02	0.85 ± 0.02	0.87 ± 0.01

Across all three datasets, the proposed method consistently outperforms the baseline models, showcasing its robustness and versatility in dealing with different types of text. The enhancements in precision, recall, and F1-scores across various datasets underscore the effectiveness of leveraging syntactic feature differences for grammar correction. As shown in Table 7, it presents the test results of the model’s response time.

**Table 7:** Test of model response time.

Dataset	GECToR	BERT-GC	NMT	Proposed
Time Taken (seconds)	CoLA: 45.3	LCoLE: 48.2	FCE: 46.8	CoLA: 38.5
				LCoLE: 40.1
				FCE: 39.6
	CoLA: 45.7	LCoLE: 48.6	FCE: 47.2	CoLA: 38.2
				LCoLE: 40.3
				FCE: 39.4

The two charts show a comparison of the performance of different methods on three datasets in terms of Error-specific Accuracy and Correction Efficiency.

**Fig 2:**
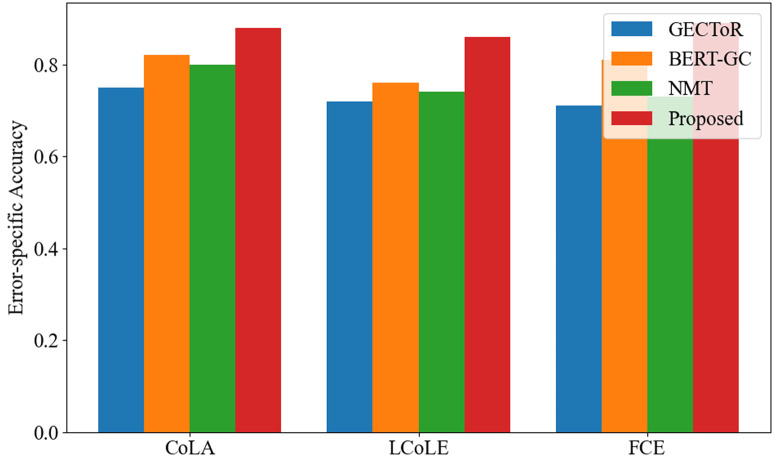
Error-specific accuracy comparison results.

In Fig 2, the proposed method shows superior performance compared with the other three methods on all datasets. For FCE datasets, the accuracy rate of this method is significantly higher than other methods, which highlights its great advantages in identifying and correcting specific types of grammatical errors. This shows that the proposed method can capture and correct these errors more accurately. The reason is that the proposed method constructs a set of sophisticated mechanisms to analyze the syntactic and semantic complexity of the text in detail. It explores the sentence structure deeply, which can not only detect superficial grammatical errors, but also dig out those more hidden and context-dependent errors. For example, when the subject-predicate agreement error is involved in a complex sentence structure, other methods may fail because of their limited analytical ability, but this method can accurately locate it.

In addition, BERT-GC performs better than GECToR and NMT, which can be attributed to BERT’s excellent deep context understanding ability. BERT can make full use of his pre-training knowledge of language patterns and semantic relations, thus improving the accuracy of error identification and correction.

**Fig 3:**
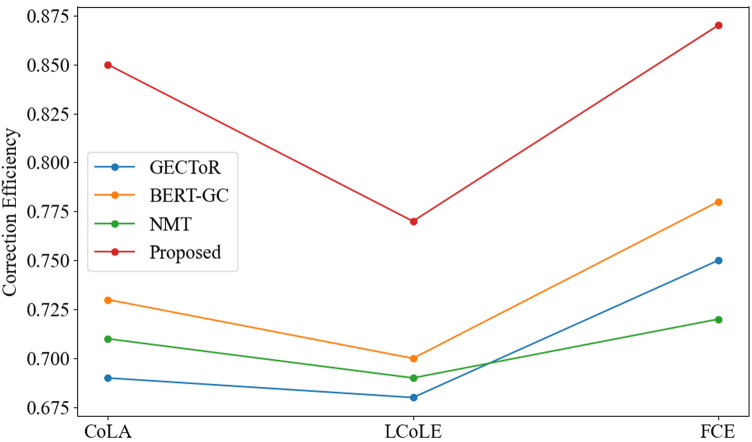
Correction efficiency comparison results.

In Fig 3, it shows that the proposed method achieves the highest efficiency on all datasets. This efficiency is not only reflected in its ability to accurately identify and correct errors, but also reflects the efficiency of minimizing the impact on the original meaning during the correction process, effectively maintaining the integrity and consistency of the text. The proposed method adopts an intelligent strategy to carefully weigh the impact of each revision on the overall coherence and integrity of the text. For example, it can avoid over-revision and lead to misinterpretation of the author’s original intention. The line chart in Fig 3 further shows that although the correction efficiency of each method fluctuates on different data sets, the proposed method always maintains a relatively high efficiency level. This stability strongly proves its robustness and wide applicability, that is, it can adapt to the characteristics of different data sets and maintain excellent performance.

The key difference between Figs 2 and 3 is that Fig 2 focuses on the accuracy of each method in identifying and correcting specific types of grammatical errors, and presents the accuracy of each method in detecting and repairing these errors in detail. On the contrary, Fig 3 focuses on how to ensure the efficiency of the original meaning while correcting errors, thus revealing the practicability and effectiveness of these methods in the actual text correction scene.

### 4.3 Ablation experiment

To verify the actual contribution of the Differential Integration (DF) mechanism to grammatical correction performance, this study designed an ablation experiment. It compared the full model with the variant model without the DF mechanism (Model w/o DF). The variant model retains only the sentence vectorization, syntactic analysis, and Seq2Seq correction modules, ignoring the computation and integration of syntactic differences between adjacent sentences. The experiment is conducted on three standard datasets: CoLA, LCoLE, and FCE. The evaluation metrics used were Precision, Recall, and F1-Score. The results are as follows:

In Table 8, the ablation experiment results indicate that the DF mechanism makes significant contributions to model performance improvement, with its core value lying in the effective capture of paragraph-level syntactic coherence. On three datasets, the complete model achieves a 5%−6% improvement in precision, recall, and F1-score compared to the variant model without DF, verifying the necessity of inter-sentence syntactic difference analysis for error detection and correction. This improvement stems from the DF mechanism’s mining of implicit rules such as topic continuity, tense consistency, and reference matching in paragraphs, enabling the model to identify cross-sentence grammatical errors (e.g., subject-verb disagreement, discourse-level reference conflicts) that are difficult to capture through single-sentence analysis. Experimental data also show that due to the lack of inter-sentence correlation analysis, the variant model has a significantly higher miss rate when processing complex sentence structures and learner paragraphs, further highlighting the advantages of the DF mechanism in simulating human cognitive logic (i.e., judging overall grammatical correctness through inter-sentence differences). This result provides empirical support for the theoretical assumptions of the DF mechanism. It also offers a new technical path for paragraph-level grammatical correction tasks. By fusing syntactic feature differences between sentences, it is possible to break through the limitations of traditional single-sentence models. This approach enables context-aware precise error correction.

**Table 8:** Results of ablation experiment.

Dataset	Type of model	Precision	Recall rate	F1 score
CoLA	Complete model	0.88	0.84	0.86
	Variant model (w/o DF)	0.83	0.79	0.81
LCoLE	Complete model	0.86	0.82	0.84
	Variant model (w/o DF)	0.81	0.77	0.79
FCE	Complete model	0.89	0.85	0.87
	Variant model (w/o DF)	0.84	0.80	0.82

## 5. Conclusion and discussion

In this paper, a novel method for grammatical correction of English paragraphs is proposed, which integrates syntactic feature difference fusion with a deep learning Seq2Seq model. The method employs BERT to capture contextual semantic representations of sentences, extracts fine-grained structural features via Stanford syntactic analysis. It also utilizes a difference fusion mechanism to systematically identify syntactic discrepancies between adjacent sentences for locating grammatical errors. Experiments demonstrate that this approach significantly outperforms existing techniques on multiple datasets, validating its effectiveness in enhancing both grammatical correction accuracy and contextual relevance.

However, this method still has limitations. The current model mainly focuses on the analysis of syntactic feature differences. Although it can effectively capture paragraph-level syntactic coherence defects, it has not fully incorporated cohesive elements (such as discourse connectives and pronominal anaphora) and pragmatic factors (such as contextual intentions and cultural backgrounds). This may lead to insufficient ability to correct errors relying on deep semantic associations (such as implicit logical contradictions and pragmatic inappropriateness). This limitation stems from the phased trade-offs in the research design. Syntactic features have clear formal definitions and computability, making them suitable as an initial entry point for paragraph-level error correction. In contrast, cohesive and pragmatic elements involve more complex semantic reasoning and world knowledge, requiring more sophisticated modeling frameworks. For example, when processing paragraphs containing discourse connectives such as “however” or “therefore”, the model may miss errors in inter-sentential semantic breaks due to a lack of understanding of logical relationships, or misjudge errors involving culture-specific expressions (such as English idioms or cross-cultural pragmatic rules).

Future research will be expanded in three aspects: First, pre-trained language models (such as GPT series) will be introduced to enhance the ability to capture discourse cohesion cues. Cohesion features (such as the logical direction of connectives and the semantic association between pronouns and antecedents) will be fused with syntactic differences through attention mechanisms. For example, semantic role labeling for connectives like “but” and “and” will be added during the encoding stage to improve the model’s understanding of paragraph logic. Second, a specialized dataset with pragmatic annotations will be constructed, and the model will be adapted by integrating situational semantics (such as argumentation logic in academic writing and politeness principles in daily conversations). For instance, special error-correction modules will be designed for the pragmatic functions of terms like “hypothesis” and “conclusion” in academic paragraphs. Third, a cross-lingual transfer learning framework will be developed to validate the universality of joint syntactic-pragmatic modeling in multilingual data. For example, the logical pair “ because-therefore” in Chinese paragraphs will be compared with “because-therefore” in English for contrastive modeling, exploring the common features of cohesion mechanisms across languages. These improvements will drive the model’s upgrade from “syntactic correctness” to “discourse coherence”, further enhancing its practical value in real-world writing scenarios.

Experimental results also show that although the model has achieved significant breakthroughs in syntactic error correction performance (e.g., an F1 score of 0.87 on the FCE dataset), there is still room for improvement in the efficiency of syntactic parsing for complex sentence structures (such as nested clauses). Follow-up research will integrate lightweight syntactic parsers and incremental computing techniques to optimize the timeliness of long-text processing, while enhancing the model’s generalization ability in low-resource scenarios (such as grammatical correction for minority languages) through adversarial training. Compared with the research of Tamilmani and Nagalakshmi (2019), this method breaks through the limitations of traditional training based on large-scale general corpora by adapting to the grammatical rules of different genres through personalized syntactic feature extraction (e.g., the rhetorical structure of literary texts and the term consistency of scientific texts). The future integration of cohesive and pragmatic factors will further strengthen this advantage. This will enable the model to more accurately capture implicit errors in domain-specific texts. For example, it can detect pragmatic irregularities in chart citations in academic papers. It can also identify contextual conflicts in character dialogues in literary works.

In summary, this paper has made an innovative stride in the field of paragraph-level grammatical correction. It not only verifies the effectiveness of inter-sentence relationship modeling through the syntactic difference fusion mechanism but also defines a technical pathway for expanding into deep semantic-pragmatic modeling. Future research will continue to optimize the model’s multi-dimensional contextual understanding capabilities to promote its wide application in fields such as automated writing assistance and intelligent education.

### 5.1 Revision description

This paper has been modified in the following parts:

1. Theoretical argumentation supplement: Add the linguistic and cognitive theoretical basis of the difference fusion mechanism in Section 3.3 to explain the rationality of syntactic differences as error signs.
2. Ablation experiment added: In Section 4.3, the ablation experiment was added to compare the model performance with or without DF mechanism, and the effectiveness of DF was proved by quantitative data.
3. Limitation discussion expansion: In the conclusion part, we admit the shortcomings of ignoring cohesion and pragmatic factors, and add some improvement directions such as introducing pre-training models and constructing special data sets in the future.

## Supporting information

S1 Data Data in figures. (XLSX)
